# Boron Removal from Mining and Synthetic Effluents by Electrocoagulation Using Aluminum Electrodes

**DOI:** 10.1155/2019/3746964

**Published:** 2019-05-09

**Authors:** Douglas Mark Lopes da Silva, Maria Tereza W. D. Carneiro, Josimar Ribeiro

**Affiliations:** Departamento de Química, Universidade Federal do Espírito Santo, 29075-910 Vitória, ES, Brazil

## Abstract

The efficiency of the electrocoagulation method to remove boron from synthetic and mining effluents was investigated in this study. Different parameters were tested using boric acid solution and effluent collected from a mining company located in the city of Vitória-ES. The results showed a percentage of boron removal of over 60% for the synthetic and mining effluents, using aluminum electrodes, pH 7.5, current density of 14.82 mA cm^−2^, and supporting electrolyte of 0.200 mol L^−1^. The electrocoagulation and chemical coagulation methods were also compared, in which the percentage obtained by electrocoagulation was 56.30% higher for the mining effluent. Thus, electrocoagulation was more efficient in boron removal, especially when appropriate parameters are applied.

## 1. Introduction

Boron is an important micronutrient for plants and is found in nature in the form of boric acid and borate salts. This shows a wide application, such as in the manufacture of borosilicate glasses, detergents, cosmetics, and fertilizers. [1] However, boron in high concentration shows toxic effects to the human organism, for example, damage to the reproductive and nervous systems. Thus, the World Health Organization (WHO) sets maximum limits of boron of 0.3 mg L^−1^ for drinking water [2]. The National Environmental Council (Conselho Nacional de Meio Ambiente, CONAMA) sets a limit of 0.5 mg L^−1^ for drinking water and a limit of 5.0 mg L^−1^ to effluents. [3]

Therefore, it is necessary to treat effluents containing boron so that it does not cause damage to the environment. There are several methods to remove boron from a system, among them the electrocoagulation (EC) method. EC is an electrochemical method of waste treatment patented in 1909 in the United States [4], in which a potential difference is applied to metal electrodes immersed in an electrolytic solution, which, under the action of an electric current, produces coagulant species* in situ*. Thus, the coagulating agent is formed by oxidation of the electrode which, simultaneously, also form hydroxyl ions and gases. The oxidized metal together with the hydroxyl ions form metallic hydroxides that will act as coagulants in the system. [4, 5]

The produced coagulant increases the ionic strength of the system, neutralizing the surface charges of the contaminating particles, destabilizing them and, consequently, allowing them to approach and start the flocculation process. This process consists in the agglomeration of the neutralized colloidal particles, in which there is an increase in the size of the flocs due to van der Waals forces between them, resulting in a sedimentation process. After that, sedimentation of the contaminating particles can be removed from the system by filtration [5, 6]. During EC, gases are also formed, and they are adsorbed on the surface of the contaminating particle, assisting in the removal process by flotation, in which the particles remain on the surface of the system [4, 7].

In EC, many metals can be used, but the most used are aluminum and iron due to high efficiency and low cost. EC reactions using aluminum electrodes are demonstrated by the following equations and the standard reduction potential (versus SHE, standard hydrogen electrode) at 298 K and 1 atm [1].

Anode:

(1) 2  AlS⇋2  Al3+aq+6  e-E°=+1.662 V

(2) 3  H2Ol⇋32  O2g+6  H+aq+6  e-E°=−1.229 V

Cathode:

(3) 12  H2Ol+12  e-⇋6  H2g+12  OH-aqE°=−0.828 V

Global:

(4) 2  AlS+9  H2Ol⇋2  AlOH3aq+32  O2g+6  H2g

Another species may arise from the hydrolysis reaction of the ion Al^3+^ during the EC process as the pH of the system increases, for example, Al(OH)^2+^, Al(OH)_2_^+^, and Al(OH)_4_^−^. The Al(OH)_4_^−^, known as tetrahydroxoaluminate, is an anion predominant at pH greater than 10.0 and water-soluble. The formation of this anion in the system impairs the flocculation process; thus, it is necessary to control the pH of the system during EC [2]. In addition to pH control, other parameters may also contribute to process optimization, such as current density, supporting electrolyte concentration, and EC time [4, 8]. Thus, Kartikaningsh et al. (2016) [1], using boron concentration of 100 mg L^−1^, current density of 2.5 mA cm^−2^, pH 8.0, and NaCl as supporting electrolyte, obtained about 90% of boron removal. Similarly, Sari and Chellam (2015) [9] obtained approximately 60% of boron removal when using a system with a current density of 50 mA cm-2 and pH 8.0. Thus, EC has the advantages of low secondary waste production, low operating costs, formation of larger and more stable flakes, and the production of gases that aid the removal of waste by flotation. [5] In this way, this research article aimed to remove boron in a synthetic effluent and in a mining effluent by EC method using aluminum electrodes, as well as the analysis of the method against pH variation, concentration of the supporting electrolyte, current density, and EC time. In this research, the efficiency of the EC method was also compared with the chemical coagulation method (CQ) using aluminum sulfate as coagulant.

## 2. Experiment

### 2.1. Electrocoagulation Cell

The cell used for EC was made of acrylic with 8 mm of thickness and sizes from 17.1 cm x 17.1 cm x 17.1 cm, with a total volume of 5* L*, with gas outlet valves and electrodes projected on the cap by screw connection. As power source, a DC Power Supply INSTRUTHERM FA3003 was used, with potential from 0 to 30* V *and current from 0 to 3.0* A*. The electrodes were prepared in aluminum alloy 1100 (Metalthaga), with an aluminum percentage of 99%. The dimensions of the plates immersed in solution were 10 cm x 10 cm x 0.2 cm, thus presenting a surface area of 208 cm^−2^ and a pair of electrodes in the reactor. At start of each experiment the electrodes were sanded with silicon carbide sandpaper of 220 and 600 grades (3M).

### 2.2. Electrocoagulation of the Synthetic and Mining Effluents

The synthetic effluent was prepared from boric acid (99%, Neon) and sodium nitrate (99%, Dinâmica) was used as supporting electrolyte. To reach 4 L of volume, it was completed with deionized water from the water purifier Sartorius Arium®, obtaining a 200 mg L^−1^ boron solution and supporting electrolyte with concentrations of 0.100 mol L^−1^, 0.200 mol L^−1^, and 0.400 mol L^−1^. On the other hand, the mining effluent was collected in the treatment plant of a mining company of the city of Vitória and the characteristics of the effluent of the mining company are presented in Table 1.

The EC of the synthetic effluent was carried out varying some parameters, such as current density in 4.81 mA cm^−2^ and 14.42 mA cm^−2^, electrolysis duration of 30, 60, 90, and 120 minutes, supporting electrolyte concentration and pH control in the range of 7.5 and 13.0. The samples with pH control were made in 120 minutes and electrolyte with concentration of 0.200 mol L^−1^, adding HCl 1.000 mol L^−1^ for adjustment of the pH. After obtaining the boron removal results and the operational cost for the synthetic effluent, the best parameters were chosen for the mining effluent. Thus, for the mining effluent, a duration of 90 minutes was used, applying current density of 14.42 mA cm^−2^ and using potassium chloride solution (99%, Dinâmica) at the concentration of 0.400 mol L^−1^ as supporting electrolyte.

### 2.3. Jar Test

To compare the EC and CQ results, jar tests were performed. This test consisted of CQ with the addition of hydrated aluminum sulfate (98%, Dinâmica) as a coagulating agent. The tests were performed in a Jar Test (Ethik Technology©), model 218-6LDB, using the coagulant mass corresponding to that produced in the EC, previously calculated by Faraday's equation (5).

(5) $$\begin{array}{c} m=i.t.\frac{M}{nF} \end{array}$$

In which* i* is the current (*A*), t is time (*s*), M is the molar mass (g mol^−1^), n is the quantity of electrons (*mol*), and F is the Faraday's constant with a value of 96485 C mol^−1^ [10].

The coagulation times for the synthetic effluent were 30 minutes and 60 minutes. The 30-minute experiment was divided into 5 minutes at 150 rpm, 10 minutes at 20 rpm, and 15 minutes at rest; and the 60-minute one was divided into 5 minutes at 150 rpm, 25 minutes at 20 rpm, and 30 minutes at rest. For the mining effluent, the duration used was 90 minutes, divided into 5 minutes at 150 rpm, 25 minutes at 20 rpm, and 60 minutes at rest. The pH of the system was corrected to 8.0 using a sodium hydroxide solution of 1.000 mol L^−1^.

All samples produced by EC and CQ were analyzed by ICP-MS (Inductively Coupled Plasma Mass Spectrometry), in which a Nexlon 300D Perkin Elmer equipment was used, with a radiofrequency power of 1500.00 W, nebulization gas flow of 0.98 L min^−1^, auxiliary gas 1.200 L min^−1^, and plasma gas 16.00 L min^−1^, with sample aspiration rate of 0.500 mL min^−1^.

### 2.4. Operational Cost

The energy consumption (CE) of the electrolytic cell and the operational cost (CO) for the EC were estimated by (6) and (7) [11].

(6) CE=i.U.tv

(7) CO=aCE+bME+cES

In which* i* is the current (*A*), U is the voltage (*V*), t is time (minutes), and V is the volume of the solution (*L*) [10]. For the operational cost, ME is the consumed mass of electrodes (*g*), ES is the mass of the supporting electrolyte (*g*), and coefficients a, b, and c are the cost (US$) of energy, aluminum, and supporting electrolyte, respectively [11]. The experimental data are shown in Table 2.

In order to calculate the energy cost, data from the EDP Espírito Santo Distribuição de Energia S.A were used, in which the cost per kWh was US$ 0.127/kWh. For sodium nitrate, aluminum mass, and potassium chloride, the cost was US$ 4.14/kg, US$ 8.05/kg, and US$ 7.81/kg, respectively. To calculate the operational cost of CQ, the respective masses of 6.210* g*, 12.330* g, *and 18.618* g* of aluminum sulfate were used for the durations of 30, 60, and 90 minutes, respectively, and the cost was US$ 20.49/kg. The mass of sodium hydroxide used for pH correction was 3.6 g per jar, with a cost of US$ 24.39/kg.

## 3. Results and Discussion

Table 3 shows the results obtained by ICP-MS of EC and CQ for the synthetic and mining effluents regarding different parameters such as pH, supporting electrolyte concentration, current density, and EC time.

### 3.1. Boron Removal as a Function of Electrocoagulation Time in the Synthetic Effluent

**Figure 1:**
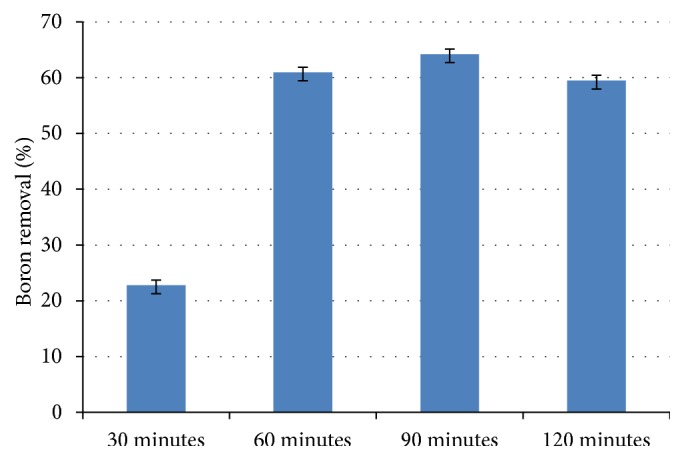
Percentage of boron removal as a function of EC time, using current density of 14.42 mA cm^−2^ and supporting electrolyte at the concentration of 0.200 mol L^−1^.

An increase in boron removal was observed when EC time was increased (30, 60, and 90 minutes) using a current density of 14.42 mA cm^−2^ and supporting electrolyte at a concentration of 0.200 mol L^−1^ (Table 3). In the 120-minute test, boron removal was reduced. Such data are shown in Figure 1.

**Figure 2:**
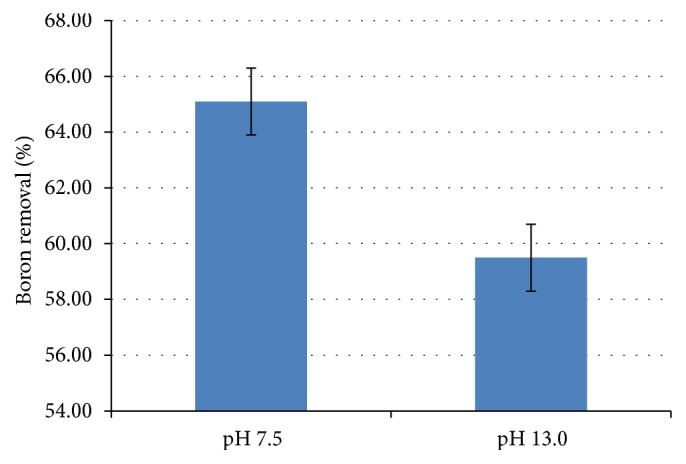
Percentage of boron removal by EC using current density of 14.42 mA cm^−2^, supporting electrolyte 0.200 mol L^−1^ and duration of 120 minutes for two pH (7.5 and 13.0), in which pH control was performed with addition of HCl 1.000 mol L^−1^.

According to Faraday's equation (5), electrolysis time is directly proportional to the produced mass of coagulant [10]. Thus, with increasing electrolysis duration, the amount of ions Al^3+^ and OH^−^ formed from electrode oxidation increases. These species in aqueous media form aluminum hydroxide which acts as a coagulant in the system [11, 12]. However, with the increase in the formation of aluminum hydroxide, the pH of the system also increases. At high pH (pH>10) the coagulant species are negatively charged to Al(OH)_4_^−^. This species is soluble, which decreases the formation of flakes formed and, consequently, decreases the removal of boron. In this way, in the duration of 120 minutes, ion Al(OH)_4_^−^ was formed due to the increased pH, reducing the percentage of removal of boron. [10, 13, 14] Thus, it was necessary to control the pH by adding HCl 1.000 mol L^−1^. The results obtained can be observed in Figure 2 for the pH of 7.5 and 13.0.

In Figure 2, an increase is observed in the percentage of boron removal at pH = 7.50 when compared to pH = 13.0, because, in this pH, there is no formation of the water-soluble species Al (OH)_4_^−^.

### 3.2. Boron Removal as a Function of the Supporting Electrolyte Concentration in the Synthetic Effluent

**Figure 3:**
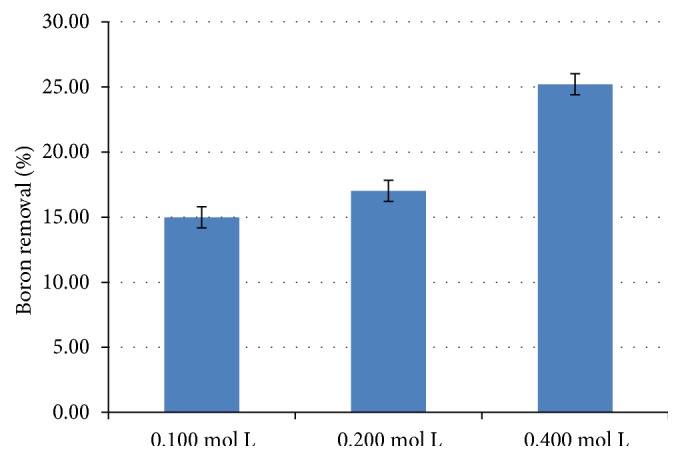
Percentage of boron removal versus variation in supporting electrolyte concentration at 0.100, 0.200, and 0.400 mol L^−1^, applying current density of 14.42 mA cm^−2^ and EC time of 30 minutes.

The supporting electrolyte, when added at high concentrations, can give the solution and interface metal-solution properties, which, in general, results in the maintenance of the high and constant ionic strength of the solution. The supporting electrolyte shows its optimized efficiency when the concentration is 100 times greater than the concentration of the electroactive species of the solution [13–15].  Figure 3 shows an increase in the percentage of boron removal with the increased supporting electrolyte used.

Thus, the increase in electrolyte concentration of 0.100 to 0.400 mol L^−1^ decreased the resistance of the solution, increasing the percentage of boron removal in the system and reducing energy cost, as shown by the result.

### 3.3. Boron Removal as a Function of Current Density in the Synthetic Effluent

**Figure 4:**
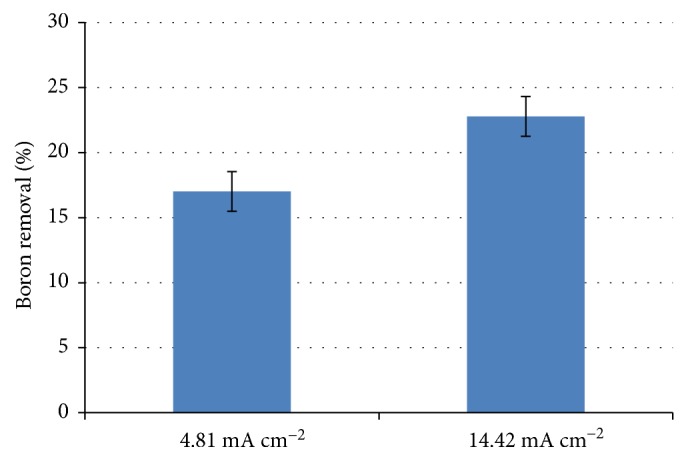
Percentage of boron removal at the current densities of 4.81 and 14.42 mA cm^−2^, with an EC time of 30 minutes and supporting electrolyte of 0.200 mol L^−1^.

As already shown by (5), the applied current is directly proportional to the mass of ions Al^3+^ produced in the system. Thus, the increase in applied current density increases the production of coagulating agent in the system [10]. Such a result can be seen in Figure 4, where increasing the current density from 4.81 mA cm^−2^ to 14.42 mA cm^−2^, for an EC time of 30 minutes, increased boron removal.

Although an increase in the boron removal is observed with increasing current density, when considering the standard deviation, such increase was not as significant. This is due to the increase in the formation of aluminum oxide that accompanies the formation of the coagulant. This is deposited on the surface of the electrode in a process called passivation, in which there is an increase in the resistance of the system and decrease in the efficiency of the method. Thus, despite increasing current density, a significant percentage difference in boron removal was not observed [16]. An alternative to reduce the passivation of electrodes is the addition of soluble anions with aluminum, such as chlorides and nitrates, because such species aid in the solubilization of the formed oxide. Such evidence can be observed in Figure 3, in which the increase in NaNO_3_ concentration increased boron removal. These results agree with the literature data [16, 17].

### 3.4. Comparison between the Percentage of Boron Removal in the Electrocoagulation and Chemical Coagulation in the Synthetic and the Mining Effluents

**Figure 5:**
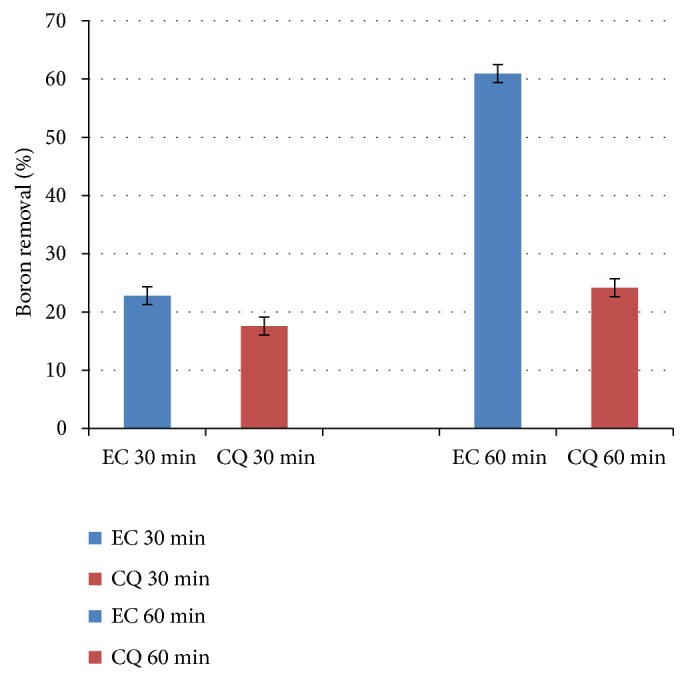
Boron removal by EC using supporting electrolyte of 0.200 mol L^−1^ and current density of 14.42 mA cm^−2^ and by CQ using Al_2_(SO_4_)_3_, for 30 and 60 minutes.

When EC was performed at 30 and 60 minutes, with current density of 14.42 mA cm^−2^ and supporting electrolyte 0.200 mol L^−1^ for the synthetic effluent, a higher boron removal was observed for EC when compared to the CQ process using aluminum sulfate as coagulant (Figure 5). It was also possible to observe a significant increase in the percentage of boron removal when time was increased from 30 to 60 minutes.

During the EC process, coagulating agent and gases* in situ* are formed. The coagulating agent produced is insoluble in water and, when adsorbed on the surface of the contaminant, it forms flocs in a process called flocculation [5, 10]. The gases produced* in situ* contribute to boron removal by flotation, in which the gaseous molecules interact with the contaminating particles, which remain suspended in the system [10, 18]. In CQ, on the other hand, the coagulating agent, in this case aluminum sulfate, is added to the system and, from the hydrolysis of the salt added, aluminum hydroxide is formed, as demonstrated by

(8) Al2SO43aq⇋2Al3+aq+3SO42-aq

(9) 2Al3+aq+6H2Ol⇋2AlOH3aq+6H+aq

The formed ions H^+^ decrease the pH of the system, making it difficult to form aluminum hydroxide, thus producing less coagulant. Therefore, EC is an advantageous method when compared to CQ, because in addition to the production of the coagulant agent* in situ*, gases are formed, which act in the removal of boron by flotation, increasing the production of these species along with EC time, as it can be observed in Figure 5. Similar behavior has been also observed by others [5, 10].

**Figure 6:**
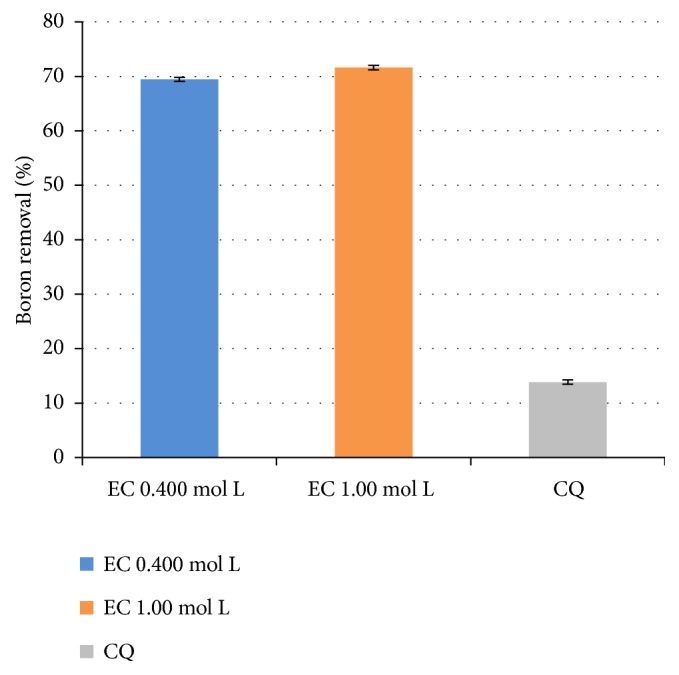
Percentage of boron removal from the mining effluent by EC for 90 minutes, current density of 14.42 mA cm^−2^ and supporting electrolyte concentration of 0.400 mol L^−1^ and 1.00 mol L^−1^, and by CQ for 90 minutes using aluminum sulfate as coagulant.

Thus, in the mining effluent, Figure 6 also shows a higher percentage of boron removal by the EC process compared to the CQ method, using the effluent from a mining company of 90 minutes, current density of 14.42 mA cm^−2^, and supporting electrolyte concentration of 0.400 mol L^−1^ and 1.00 mol L^−1^.

The results shown in Figure 6 and Table 3 indicate that the electrocoagulation at both supporting electrolyte concentrations left the effluent of the mining company within the parameters required by resolution of CONAMA n° 396/2008 of 5.00 mg L^−1^. Thus, the effluent from the mining company after electrocoagulation presents satisfactory boron levels. When one compares the results with the literature, for example, Wided et al. [17], using a current of 86.44 mA.cm^−2^, for 60 minutes and with boron concentration of 5 mg L^−1^, the authors obtained a 23% higher boron removal in EC when compared to CQ. Kartikaningsih et al. [1], using a current of 2.5 A, pH = 8.0, for 180 minutes and boron concentration of 100 mg L^−1^, obtained a percentage of boron removal 18% higher for EC when compared to CQ.

### 3.5. Operational Cost of EC and Comparison with Chemical Coagulation

The operational cost was calculated based on (6) and (7) and with the data listed in Table 2. Thus, Table 4 shows the energy and operational costs of the EC process for the synthetic and mining effluents.

In Table 4, it can be observed that increasing the time from 30 to 60 minutes increases the operational cost; however, as shown in Figure 1, the percentage of boron removal has a significant increase when duration goes from 30 to 60 minutes, which shows a more satisfactory result because the increase in cost was small.

Ezechi et al. [19], treating 1.0* L* of synthetic effluent containing boron at a concentration of 20 mg L^−1^ during a 60-minute EC time and a current density of 12.5 mA cm^−2^, obtained an operational cost of US$ 0.22. According to the author, an operational cost considered satisfactory should be less than US$ 1.27. Thus, the results obtained for the synthetic and mining effluents were satisfactory.

Table 5 shows the comparison between the operational cost of EC and CQ, of 60 minutes for the synthetic effluent, and 90 minutes for the mining effluent.

The results demonstrate that EC is an important method for waste treatment, since it was more efficient and advantageous for both effluents. In addition to its lower operational cost, a significant increase in boron removal was also obtained.

## 4. Conclusion

The results showed that the use of EC was an important method for the removal of boron in synthetic effluent and mining effluent in the mining company, where a boron removal of more than 60% was observed in both effluents, and became the effluent of the mining company have levels of boron below the maximum allowed by CONAMA. A low operational cost was also observed, not exceeding US$ 0.20 for the synthetic effluent and US$ 0.303 for the mining effluent. When comparing EC and CQ, the electrochemical method proved to be more advantageous, because in addition to a lower operational cost, a significant increase in the percentage of boron removal was also obtained. The results also showed that the variation of parameters such as pH, current density, supporting electrolyte concentration and EC time may contribute to a higher boron removal, a fact observed for the synthetic effluent. Thus, EC is an important technique that can be applied in the removal of industrial waste and can be used to reduce environmental impacts caused by the release of these effluents into rivers and soil, avoiding risks to the environment and human health.

## Figures and Tables

**Table 1:** Characteristics of the effluent collected in the STS (Sawage Treatment Station) of the mining company.

Composition	Concetration (mg L^−1^)
Boron	16.22
Total carbon	181.0
Phenols index	0.053
Phosphor	72.50
Total nitrate	0.700
Total nitrite	0.309
Ammoniacal nitrogen	7.100
Total nitrogen	14.00
Surfactants	0.128
Total suspended solids	150.0
Total fixed solids	55.00
Volatile suspended solids	95.00
Total oils and greases	146.0
BOD*∗*	262.0
COD*∗∗*	1910.0

*∗* Biochemical oxygen demand; *∗∗* COD: chemical oxygen demand

**Table 2:** EC data to estimate the operational cost of synthetic effluent for 30, 60, 90, and 120 minutes using a current (i) of 3.0 A and supporting electrolyte (ES) of 0.200 mol L^−1^, and for the mining effluent, for 90 minutes, using 3.0 A of current and 0.400 mol L^−1^ of supporting electrolyte.

Effluent	Time (min)	Potential (V)	i (A)	Vol. (L)	ES (g)	CE (Wh L^−1^)	ME (g)
Synthetic	30	12.6	3.0	4.0	68.00	4.725	0.503
Synthetic	60	12.5	3.0	4.0	68.00	9.375	1.006
Synthetic	90	11.7	3.0	4.0	68.00	13.162	1.510
Synthetic	120	12.8	3.0	4.0	68.00	19.200	2.013
Mining	90	3.5	3.0	4.0	29.80	3.937	1.510

**Table 3:** Results obtained for the electrocoagulation and chemical coagulation tests for a synthetic effluent with initial boron concentration of 200 mg L^−1^ and for the effluent of mining company with initial boron concentration of 16.220 mg L^−1^.

Effluent	Test	[ES] mol L^−1^	pH	j (mA cm^−2^)	Time (min)	[B] mgL^−1^	Boron removal (%)
Synthetic	EC	0.400	8.4	14.42	30	149.57	25.2
Synthetic	EC	0.100	8.7	14.42	30	170.03	15.0
Synthetic	EC	0.200	7.8	4.81	30	165.96	17.0
Synthetic	EC	0.200	8.5	14.42	30	154.42	22.8
Synthetic	CQ	-	8.0	-	60	151.71	24.1
Synthetic	CQ	-	8.0	-	30	164.86	17.6
Synthetic	EC	0.200	10.7	14.42	60	78.07	60.9
Synthetic	EC	0.200	11.9	14.42	90	71.52	64.2
Synthetic	EC	0.200	13.1	14.42	120	81.00	59.5
Synthetic	ECpH	0.200	7.5	14.42	120	69.80	65.1
Mining	EC	0.400	7.5	14.42	90	4.93	69.5
Mining	EC	1.000	7.5	14.42	90	4.58	71.6
Mining	CQ	-	8.0	-	90	14.54	13.9

ECpH = electrocoagulation with pH control at 7.5; j = current density; [ES] = supporting electrolyte concentration; EC = electrocoagulation; CQ = chemical coagulation

**Table 4:** Energy and operational costs for EC, for the synthetic effluent, performed for 30, 60, 90, and 120 minutes, current density of 14.42 mA cm^−2^ and supporting electrolyte 0.200 mol L^−1^, and for the mining effluent with a current density of 14.42 mA cm^−2^, for 90 minutes and supporting electrolyte 0.400 mol L^−1^.

Effluent	Time (min)	Energy cost (US$)	Operational cost (US$)
Synthetic	30	0.00060	0.187
Synthetic	60	0.00120	0.191
Synthetic	90	0.00167	0.196
Synthetic	120	0.00244	0.200
Mining	90	0.00050	0.245

**Table 5:** Comparison of operational cost in the electrocoagulation (EC) and chemical coagulation (CQ), during 60 minutes for synthetic effluent and, for mining effluent, 90 minutes, using current density of 14.42 mA cm^−2^ in the EC.

Effluent	EC-cost (US$)	CQ-cost (US$)	Boron removal EC (%)	Boron removal CQ (%)
Synthetic	0.191	0.343	60.9	24.1
Mining	0.245	0.469	69.0	12.7

## Data Availability

The data used to support the findings of this study are available from the corresponding author upon request.
